# Individual’s Social Perception of Virtual Avatars Embodied with Their Habitual Facial Expressions and Facial Appearance

**DOI:** 10.3390/s21175986

**Published:** 2021-09-06

**Authors:** Sung Park, Si Pyoung Kim, Mincheol Whang

**Affiliations:** 1 School of Design, Savannah College of Art and Design, Savannah, GA 31401, USA; 2 Department of Emotion Engineering, Sangmyung University, Jongno-gu, Seoul 03016, Korea; 201731114@sangmyung.kr; 3 Department of Human Centered Artificial Intelligence, Sangmyung University, Jongno-gu, Seoul 03016, Korea; whang@smu.ac.kr

**Keywords:** virtual avatar, virtual human, virtual character, embodied conversational agent, social interaction, empathy

## Abstract

With the prevalence of virtual avatars and the recent emergence of metaverse technology, there has been an increase in users who express their identity through an avatar. The research community focused on improving the realistic expressions and non-verbal communication channels of virtual characters to create a more customized experience. However, there is a lack in the understanding of how avatars can embody a user’s signature expressions (i.e., user’s habitual facial expressions and facial appearance) that would provide an individualized experience. Our study focused on identifying elements that may affect the user’s social perception (similarity, familiarity, attraction, liking, and involvement) of customized virtual avatars engineered considering the user’s facial characteristics. We evaluated the participant’s subjective appraisal of avatars that embodied the participant’s habitual facial expressions or facial appearance. Results indicated that participants felt that the avatar that embodied their habitual expressions was more similar to them than the avatar that did not. Furthermore, participants felt that the avatar that embodied their appearance was more familiar than the avatar that did not. Designers should be mindful about how people perceive individuated virtual avatars in order to accurately represent the user’s identity and help users relate to their avatar.

## 1. Introduction

Humans communicate with others via verbal and non-verbal communication. Through dyadic social interaction, people elicit the other’s intention and emotion [1]. Facial expressions represent non-verbal communication channels [2]. The face is the most recognizable region and has unique characteristics that represent an individual [3]. Humans are born with an innate capability to sense and perceive the most important person (i.e., mother) at the early stage of life. Infants are known to discriminate facial features starting at two months after birth [4], and they also prefer facial features over other shapes and forms [5]. Hiding one’s face implies the concealment of one’s identity. For example, covering a face with a mask may be considered negative social behavior [6].

The rapid advancement of VR (Virtual Reality) technology facilitates the introduction of expressive services tailored to the metaverse. Virtual experiences using HMD (Head-Mounted Display) are now prevalent in households due to video games. In addition, the AR (Augmented Reality) industry is growing through mobile platforms with the availability of engaging entertainment services. Naturally, virtual avatars, a conduit that connects the virtual world to the user, have gained much attention. Many users are interested in projecting or extending their identities through avatars in the internet’s social landscape.

There are various ways to express oneself through a virtual avatar. The most direct way is to apply one’s physical characteristics to an avatar that embodies the user’s facial appearance or proportions [7]. Studies are also considering the application of a user’s habitual expressions based on facial muscle movement [8]. A virtual avatar with the user’s unique signature may elicit social responses such as perceived similarity and familiarity.

### 1.1. Habitual Facial Expressions and Facial Appearance

The human face consists of 20 facial muscles. Humans communicate through an interplay of these muscles, which produce expressions. Facial expressions enable social communication, which abides by shared rules [9]. They are a powerful source of visual information that embodies the individual’s emotions, behavioral predisposition, and intention [10]. Humans can infer the interaction partner’s psychological state through facial expressions and identify their traits [11]. In psychology, an individual’s traits are, by definition, their habitual pattern of thoughts or affect.

Facial expressions are individual behavioral habits that consist of patterned muscle movement. Such patterns include unique muscle characteristics (e.g., the intensity of the movement of each facial muscle). As a result of these individual differences, people can reliably discriminate themselves from others [8].

On the other hand, facial appearance provides a person’s unique identity from the physical features, specifically face and head. Although the perception of appearance relies on many environmental factors (e.g., head pose, lighting conditions), there are descriptive characteristics of a particular individual, such as the location of the eye, nose, and mouth. In our study, we used such facial landmarks to identify critical regions of the face by defining their coordinates (x,y) on the facial image.

Visual perception plays an integral part in facial recognition, which also applies to recognizing oneself. The easiest way to look at oneself is through a mirror. Being able to recognize one’s own face is one of the critical prerequisites of self-consciousness and self-identity. Only humans and a few animals may recognize themselves through a mirror [12]. For humans, this ability develops at the age of two. This ability correlates with empathic and altruistic behavior.

Humans feel a sense of closeness to familiar entities. They also feel more intimate with objects that they are repeatedly exposed to, even without interacting with these (i.e., mere exposure) [13]. An object to which a person is familiarized through repetitive exposure may elicit positive responses [14,15]. For example, stimuli such as names [16] or photos [17] may elicit positive responses after repeated exposure. This phenomenon may also be observed with facial perception. When participants viewed a specific face repetitively, they described it as more familiar, similar, and attractive than those who did not [18].

Humans belong to social circles of varying size. Individuals have a higher chance of getting exposed to a member in the same group than to a member in a different group. When exposed to identical situations, people in the same group tend to exhibit similar responses. The more members express different responses, the lesser the probability of sustaining the group [19].

Exhibiting a similar response to an identical stimulus is related to empathy. In a dyadic interaction, an empathic response is manifested by mimicking the other’s facial expressions or gestures [20]. Sustaining a similar expression or empathic response for a long time results in the repeated utilization of the respective muscles responsible for empathic expressions. Repetitive use of certain muscles affects bone structure and as a result, leads to an appearance that is similar to that of the significant other [21].

Furthermore, perceived similarity is known to entail a positive face-to-face interaction. People are predisposed to think that in dyadic socialization, a part of their partner’s attitude, values, and beliefs is similar to theirs [22,23]. People tend to like and trust people who have a similar physical appearance more than those who do not [24].

### 1.2. Virtual Avatar

The term avatar is derived from a Sanskrit word and connotes the incarnation of a deity. In modern society, the user’s mental model of an avatar is that it is an alter ego of the user that can interact with other virtual avatars in a virtual world [25]. Recently, the need for a virtual avatar has not only come from games, movies, advertisements, and remote collaboration but has extended to medical practice and crime investigation. Research, design, and development explore the avatar model and how it can imitate users in real time. Realistic animation is possible by depicting the movement based on bone and muscle structure, considering the real-world laws of physics.

In general, the more similar the illustration of a virtual avatar is to the user, the more immersive their experience [26,27]. Nevertheless, a very realistic but imperfect depiction of a user may lead to negative feelings [28]. Virtual characteristics that reach a certain point of human likeness tend to elicit a feeling of eeriness.

Much research has been conducted on the interaction channels of virtual avatars. There has been much attention on non-verbal expressions such as the gaze, the facial expression, and gestures of an avatar. For example, minute movements of the pupil add a sense of immersion and social presence. Studies found that participants perceived a higher level of social presence when communicating via richer media than through a text-based medium [29,30,31].

In a virtual environment, users may use their virtual avatar to represent themselves. Users tend to prefer an avatar that embodies their unique and exclusive characteristics that differentiate them from the others. Some people prefer an avatar that is similar to themselves, while others prefer their avatar to be an idealized version of themselves. Users who adopted such avatars reported higher satisfaction and attachment [32]. Users are more motivated to use avatars that have a facial appearance similar to theirs than those that do not [24].

However, the majority of avatar illustrations and expressions do not consider the individual’s facial characteristics. Applying individualized facial habits or appearances does not require sophisticated technology and is viable with the current computer systems available to the mass. However, software that can animate such virtual avatars needs to be developed with investment and resources.

Another reason why individuated avatars are not prevalent involves the users. Many users do not recognize their own facial habits and would have trouble customizing the facial characteristics by themselves. It would be necessary for the application to capture and analyze the user’s facial movements and suggest a personalized avatar for approval before use. The users may feel that this is a hassle, not to mention that there is resistance from users against taking a video of their own face. Most importantly, research lacks an understanding of common elements applicable to individuated virtual avatars. Specifically, we do not clearly understand the social effects of personalized virtual avatars with individualized features. Would people prefer avatars with their appearance or habitual expressions? Would people perceive a similarity between the avatar and themselves? Would people be able to relate to the avatar and use it for their profile in a social networking service?

### 1.3. Research Goal

Humans have universally recognizable expressions. Ekman found a universal relationship between facial muscle movements and specific emotions (e.g., happiness, sadness, anger, fear, surprise, disgust, interest) [33]. Despite the universality, individual differences exist in the *intensity* of each muscle movement. Researchers also found that the asymmetrical measures of facial regions identify stable individual differences [34].

A facial habit results from a habitual personal pattern that exhibits a unique individual signature. Facial recognition based on these individual differences in expression analyzes the movement pattern of facial muscles to discriminate individuals [8].

Another factor to consider is the individual’s appearance. The perception of a form is necessary to identify an object [35]. The holistic form is a pivotal component required to distinguish an individual [36].

In summary, our research aims to evaluate the perceived social effect of a virtual avatar using two markers: (1) habitual facial expressions captured through the *intensity* of muscle movement and (2) facial appearance identified using facial *landmarks*. The research hypotheses are summarized accordingly in Table 1. We added the third hypothesis because both facial habit and facial appearance involve the facial muscle, and therefore, an interaction may occur. Thus, we intend to analyze whether facial habits (independent variable) have a different effect on the social constructs (dependent variables) depending on facial appearance (independent variable).

In short, the study aims to evaluate people’s social perception of an avatar that embodies the unique and individual characteristics of the user. We planned to investigate the interaction of the two independent variables (facial appearance, facial habit) and their respective main effects.

## 2. Methods

### 2.1. Participants

Forty-five university students were recruited as participants. The participants’ average age was 23.78 years (SD = 2.88), with 20 males and 25 females. We recommended that the participants get sufficient sleep the day before the experiment. We selected participants with a corrective vision of 0.7 or above to ensure the participants’ reliable recognition of visual stimuli. All participants were briefed on the purpose and procedure of the experiment and signed a consent form. Participants were given participation fees as compensation.

### 2.2. Materials

#### 2.2.1. Video Stimulus

The current study used a video stimulus to elicit participants’ facial responses to produce data to create an individuated avatar. We used video materials known to evoke emotions, which were empirically verified by an experiment conducted in and provided by Stanford University (*n* = 411, [37]).

For each emotional state (positive and negative), we selected two candidate stimuli from Stanford’s materials [37]. We conducted a manipulation check on all candidate materials. With regard to the positive stimuli, participants perceived the two video stimuli as positive. The results did not show a significant difference from those of the Stanford study. However, there was no significant change in the facial expression of participants when the negative stimuli were exposed. In a follow-up questionnaire, participants reported having a negative emotional state but did not display a negative facial expression. Since the current experiment requires valid participant data on emotional expression to be applied to a virtual avatar, we decided not to include stimuli evoking a negative emotional state.

#### 2.2.2. Video Analysis

**Figure 1:**
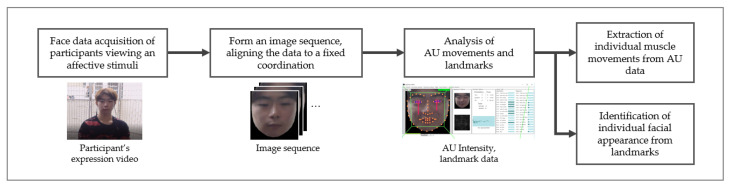
The analytical process of identifying individual muscle movements and facial appearance.

**Figure 2:**
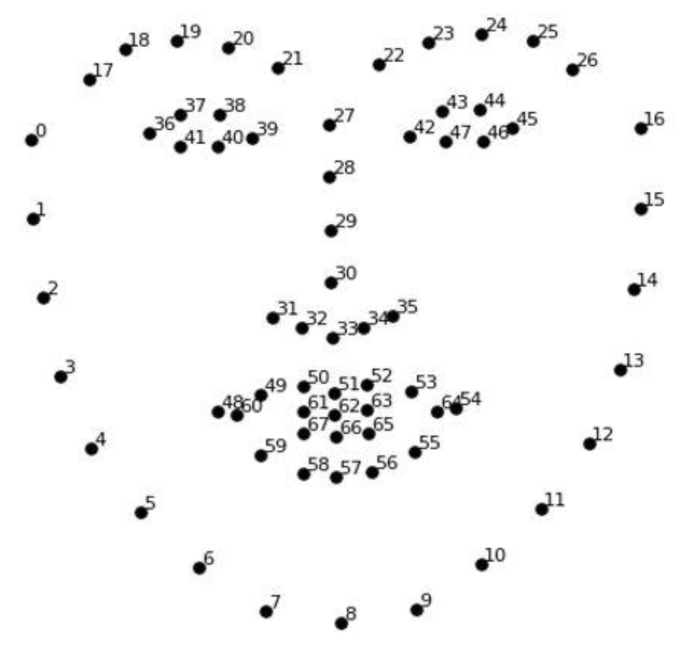
The 68 facial landmarks used to identify the participant’s facial appearance.

We used Open Face, which is open-source software that enables face recognition with deep neural networks [38]. We used AU (Action Units) as the basic unit for appraisal from the Facial Action Code System (FACS) [39]. Figure 1 depicts the process. We first normalized the facial region from the participants’ videos. The video was organized as a sequence of images of fixed size (200 × 200 pixels). From this image sequence, we elicited the intensity of AU movement and the 68 facial landmarks (see Figure 2). The landmarks extract the coordinates (x,y) of key facial regions (e.g., the eye, eyebrows, nose, lips, and chin). The movement and intensity of AU were identified from the AU vector data in HOG (Histograms of Oriented Gradients) [40]. We elicited the individual’s habitual expression data from the AU movement intensity. We elicited the individual’s facial appearance from the landmark data.

#### 2.2.3. Virtual Avatar

**Figure 3:**
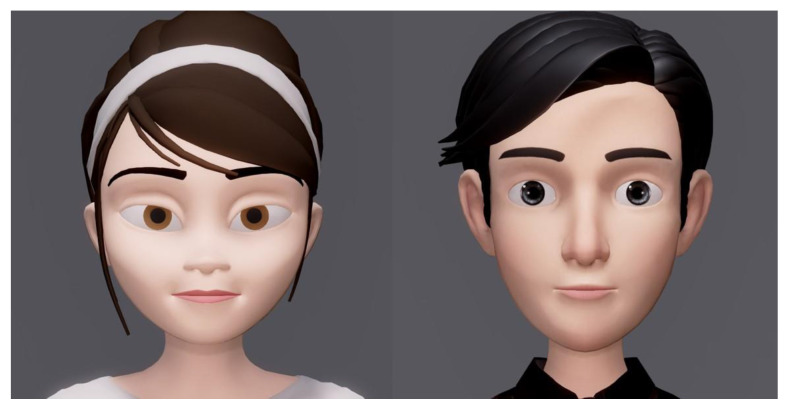
The baseline virtual avatar models in the study.

We designed two baseline avatars, male and female, to embody the participant’s expressive habits and facial appearances (see Figure 3). For the female model, we modified a public model available from an open source [41]. To visualize the muscle movement, we produced AU-based blend shapes using the animation software Maya (Autodeck). We used blend shapes that morphed the lower face of the virtual avatar for a more natural look. Table 2 shows the relationship between blend shapes and facial muscles.

**Figure 4:**
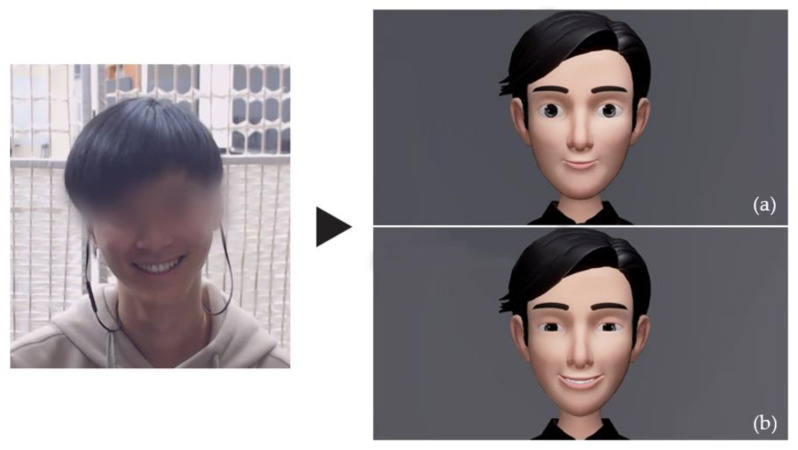
An example of the baseline virtual human morphed based on the participant’s (**a**) facial appearances and (**b**) habitual expression.

We used the Unity 3D engine to render and animate the virtual avatar [42]. Figure 4 depicts the two versions of the avatar with the participant’s facial signature (facial appearances, habitual expression) applied. How participants viewed such variations and what was measured will be explained in Section 2.3 (Experiment Procedure).

#### 2.2.4. Subjective Appraisal of Social Constructs

The current study investigated participants’ perceptions (similarity, familiarity, attraction, liking, and involvement) of virtual avatars. All constructs involve the subjective appraisal by participants rather than an objective quantitative measurement. Table 3 depicts their operational definition. Each construct was measured on a 7-point Likert scale. For example, the seven items of similarity were *slightly*, *somewhat*, and *extremely* toward both ends (dissimilar and similar) with *neutral* in the middle.

Similarity connotes the degree to which the user sees themselves as similar with the avatar. Some research includes attitudinal similarity (e.g., personality, attitude, belief system) in the definition [18,43]. However, in this study, we limited the definition to only include the physical likeliness to the participant and formulated the survey question accordingly. We purposely designed the study to eliminate interaction with the virtual avatar to investigate the effect of its mere presence without any convoluted variables that may arise from interactions. Since there is no interaction with the virtual avatar, it is extremely difficult to validly assess attitudinal similarity.

It is important to emphasize that we investigated *perceived* similarity as opposed to actual similarity. Researchers have made a clear distinction between the two constructs [44]. *Actual* similarity is measurable and quantifiable using standardized personality assessment. As the paper will discuss later, the relationship between similarity and attraction is critical. Some research studies suggest that only perceived similarity is a prerequisite to eliciting attraction [45,46,47]; other research emphasizes the importance of actual similarity [48]. In this study, mainly for consistency with other perceived constructs, we investigated the perceived similarity.

Perceived familiarity was measured to assess the degree to which participants were familiar with the virtual avatar that had the participant’s facial characteristics applied. In interpersonal and social science literature, this construct connotes “being knowledgeable” or acquainted with a person [18,49] or a concept [50,51]. That is, a priori knowledge is necessary to measure perceived familiarity. For example, in psychology, after an interaction (e.g., phone call, discussion) with a person, the participant felt subjective familiarity with the person similar to what they would feel with a close friend [49]. Other studies measured familiarity using objective quantitative measures, such as the amount of exposure to a person’s photo and not just focusing on perception [18].

Some studies use the terms perceived familiarity and resemblance (perceived similarity) interchangeably [49]; however, we measured the two constructs (perceived similarity and perceived familiarity) independently. The literature suggests that the two constructs correlate and have a causal relationship, with attraction as a mediating variable [18]. In our study, we minimized interaction with the virtual humans (e.g., conversation) to test the mere exposure effect.

Since the pioneering work of Byrne [52] (for a review of attraction as a research paradigm, see [53]), researchers have investigated interpersonal attraction in relationships [54]. Researchers widely accept Newcomb’s definition of attraction as the most comprehensive one, and it is defined as follows: “Attraction refers to any direction orientation (on the part of one person toward another) which may be described in terms of sign and intensity” (Page 6) [55].

Studies on attraction generally investigate the relationship between the independent variables (e.g., attitudinal similarity, physical attractiveness) and the attraction response as a dependent variable. It is critical to note that attraction is distinguished from attractiveness, i.e., characteristics (e.g., attractive personality, good looks) that attract others [56]. In our study, we obtained the participant’s perceived attraction (dependent variable) to the virtual avatar, which varied according to different facial features (independent variable). The intensity of attraction depends on many factors such as their relationship (e.g., parent–child, wife–husband) and the duration of interaction (e.g., long-term, first acquaintance) [57].

Perceived liking, as a construct, is defined as the degree to which the participant likes or dislikes the other person in a dyad. A causal pattern consists between the perception of being liked and liking the other [58]. Compared to attraction, perceived liking has a corresponding place on a like–dislike spectrum, whereas attraction is located on an attraction–repulsion spectrum [59].

In psychology, involvement connotes *approach* predispositions (e.g., empathy, sympathy, challenge) as opposed to distance, which refers to *avoidance* predispositions (e.g., antipathy, irritation, boredom) [24]. The two constructs are unipolar. Involvement refers to the degree to which the participants relate to and empathize with the virtual avatar. Since empathy is mainly dependent on the task and context [60,61]), we provided the context that the virtual agent would be used in a profile for a social networking service.

### 2.3. Procedure

**Figure 5:**
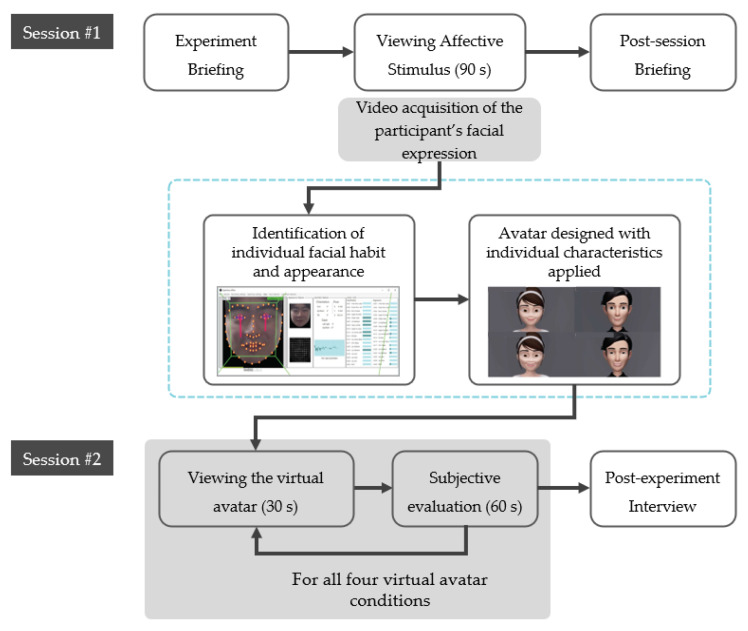
The experiment consists of two sessions, with one week in between for each participant.

Figure 5 outlines the experiment procedure. The experiment was conducted twice, with an interval of one week between the two sessions (i.e., Session #1 and Session #2).

**Figure 6:**
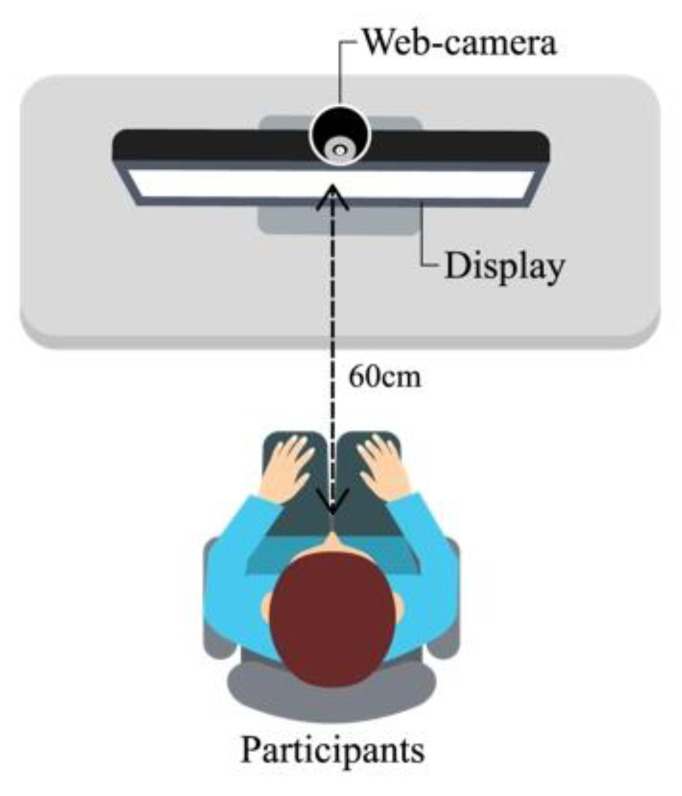
The experiment environment.

In the first experiment, the participants were briefed about the purpose of the experiment and the procedures. Then, participants viewed the two affective stimuli from the display in a relaxed position (see Figure 6). Participants were guided not to force any expression but display the natural expression felt from the viewing. The web camera on display recorded a video of the participant’s facial responses for 90 s. Then, the participants left the experiment after a brief explanation of the second experiment session.

In between the two sessions, we produced the following four virtual avatars for the second experiment session based on the data acquired from the participants:

(1) An avatar with both the habitual facial expression and appearance applied;
(2) An avatar with only the facial appearance applied;
(3) An avatar with only the habitual facial expression applied;
(4) Baseline avatar with none of the individual data applied.

For an avatar without any habitual facial expression applied (2 and 4), AU movement based on the literature was applied instead [39]. For an avatar without any facial appearance applied (3 and 4), the original baseline appearance of the avatar was used (Figure 3).

Then, the participants viewed these virtual avatar stimuli. The study used a 2 × 2 within-subject design. There were two levels of habitual expression (applied or not) and facial appearance (applied or not), respectively.

Every participant viewed all four virtual avatar types. The order of the virtual avatar was randomized using a Latin square to counter the potential learning and fatigue effect. After viewing the avatar for 30 s, the participant responded to a subjective questionnaire.

Interaction with the virtual human was limited to mere exposure as opposed to an interactive one (e.g., conversation). The strength of the subjective response was contingent on the nature of the task [62] and may have elicited a confounding effect, which would be difficult to identify.

### 2.4. Statistical Analysis

To understand the effects of the two independent variables (habitual facial expression, facial appearance), we conducted a two-way ANOVA on the participant’s subjective evaluation of the four avatars.

Data from participants who did not exhibit any facial expressions during the experiment were excluded during the acquisition process. The exclusion criteria are outlined as follows. First, we divided the non-expression interval and the expression interval. The latter was defined based on the average expression data. The intensities of AU 6 (Cheek raiser) and AU 12 (Lip corner puller) during the expression interval were compared to those of the non-expression interval. If the intensity during the expression interval was less than the non-expression interval or non-existent, we excluded the participant’s data. The Latin square factors were tested to examine whether the order affected the dependent variable. The Latin square order did not affect data, so all results were collapsed over these variables.

## 3. Results

### 3.1. Similarity

**Figure 7:**
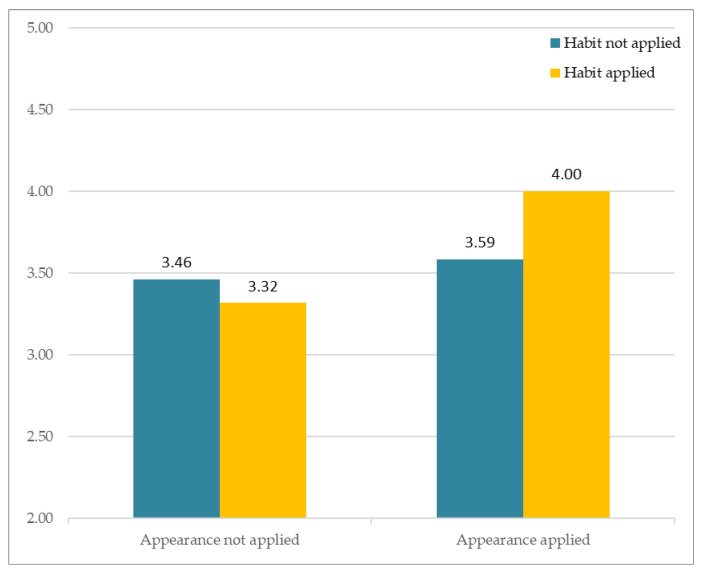
Subjective appraisal of perceived similarity.

The results of analysis of subjective perception involving similarity are as follows. Figure 7 depicts participants’ responses to the different avatars that varied according to two factors (facial habit and facial appearance). The *Y*-axis indicates the average of subjective Likert ratings. The results showed no significant interaction between Facial Habit × Facial Appearance, F(1, 163) = 2.517, *p* > 0.11. Of particular importance, the results showed that Facial Habit had a significant main effect, F(1, 81) = 5.182, *p* < 0.05. On the other hand, Facial Appearance had no significant main effect, F(1, 81) = 0.576, *p* > 0.44.

### 3.2. Familiarity

**Figure 8:**
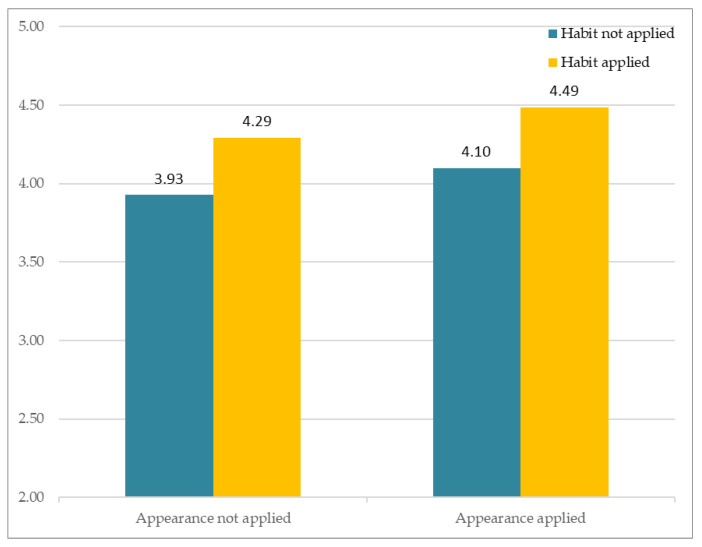
Subjective appraisal of perceived similarity.

The results of analysis of subjective perception involving familiarity are as follows. Figure 8 depicts participants’ responses to the different avatars that varied according to two factors (facial habit and facial appearance). The *Y*-axis indicates the average of subjective Likert ratings. The results showed no significant interaction between Facial Habit × Facial Appearance, F(1, 163) = 0.004, *p* > 0.94. Of particular importance, the results showed that Facial Appearance had a significant main effect, F(1, 81) = 4.182, *p* < 0.05, whereas Facial Habit had no significant effect, F(1, 81) = 0.966, *p* > 0.32.

### 3.3. Attraction

**Figure 9:**
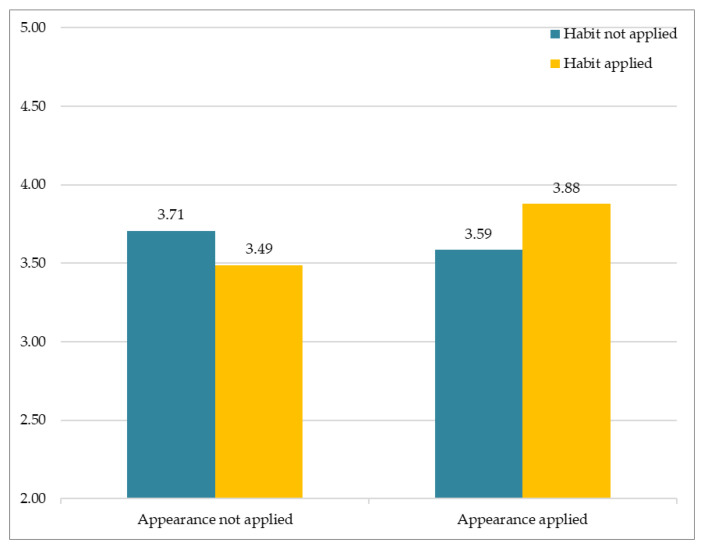
Subjective appraisal of perceived attraction.

The results of the analysis of subjective perception involving attraction are as follows. Figure 9 depicts participants’ responses to the different avatars that varied according to two factors (Facial Habit and Facial Appearance). The *Y*-axis indicates the average of subjective Likert ratings. The results showed no significant interaction between Facial Habit × Facial Appearance, F(1, 163) = 2.3, *p* > 0.13. Both Facial Appearance, F(1, 81) = 0.047, *p* > 0.82, and Facial Habit, F(1, 81) = 0.631, *p* > 0.42, had no significant main effect.

### 3.4. Liking

**Figure 10:**
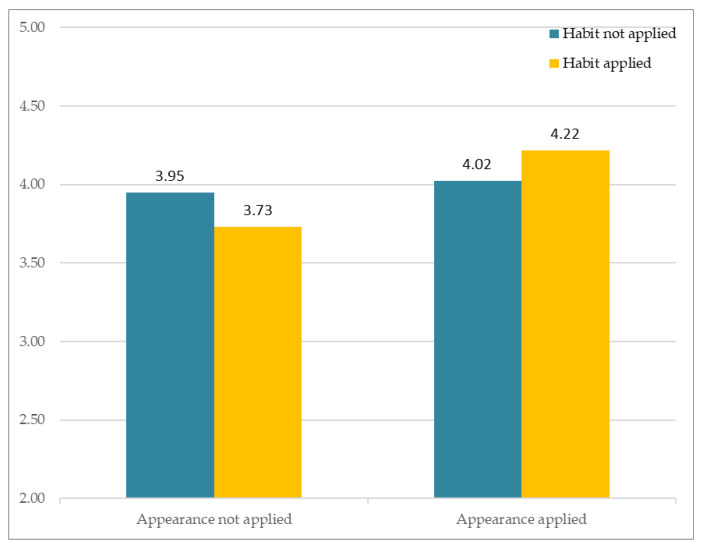
Subjective appraisal of perceived liking.

The results of analysis of subjective perception involving liking are as follows. Figure 10 depicts participants’ responses to the different avatars that varied according to two factors (Facial Habit and Facial Appearance). The *Y*-axis indicates the average of subjective Likert ratings. There was no significant interaction between Facial Habit × Facial Appearance, F(1, 163) = 1.165, *p* > 0.28. Both Facial Appearance, F(1, 81) = 0.004, *p* > 0.94, and Facial Habit, F(1, 81) = 2.133, *p* > 0.14, had no significant main effect.

### 3.5. Involvement

**Figure 11:**
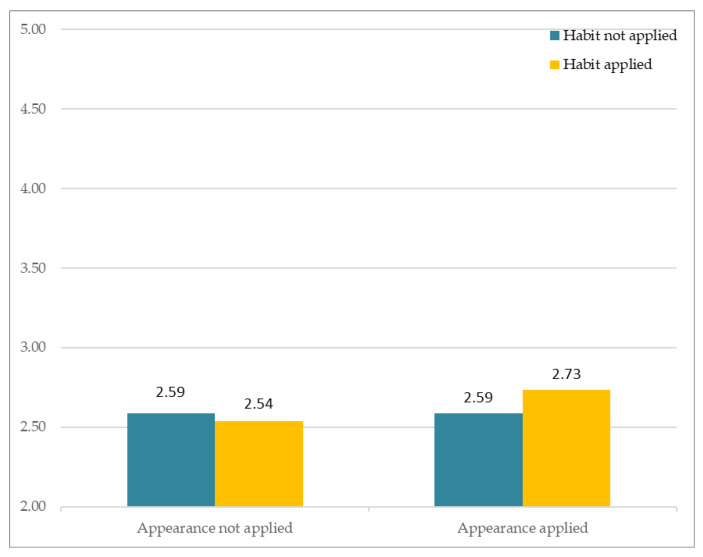
Subjective appraisal of perceived involvement.

The results of analysis of subjective perception related to involvement are as follows. Figure 11 depicts participants’ responses to the different avatars that varied according to two factors (Facial Habit and Facial Appearance). The *Y*-axis indicates the average of subjective Likert ratings. The results showed no significant interaction between Facial Habit × Facial Appearance, F(1, 163) = 0.221, *p* > 0.63. Both Facial Appearance, F(1, 81) = 0.055, *p* > 0.81, and Facial Habit, F(1, 81) = 0.221, *p* > 0.63, had no significant main effect.

### 3.6. The Correlations between Social Perceptions

**Figure 12:**
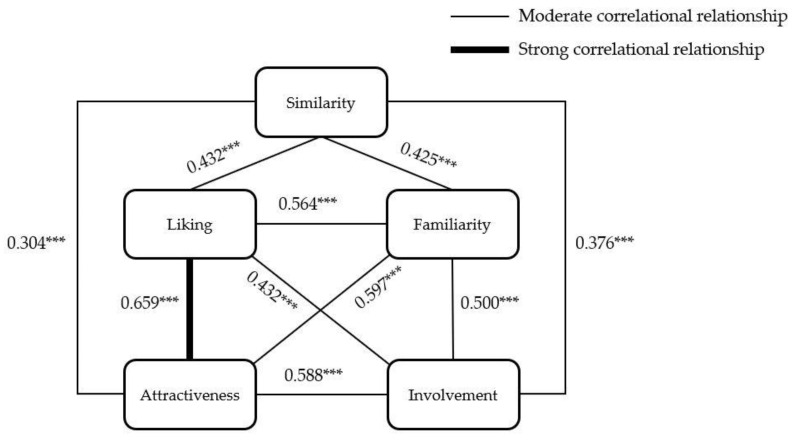
The correlational relationship between social constructs. *** *p* < 0.001

We conducted a bivariate correlation analysis to understand the relationship among participant’s social perceptions of the virtual avatars (see Table 4). The results show a significant correlation in all pairs of the analysis. The correlation between perceived attraction and liking was the highest (r = 0.695, *p* < 0.01) (see Figure 12). The implications of the correlation results will be discussed, integrating results from other analyses.

### 3.7. Data Categorization

Thus far, we identified that facial habit had a main effect on similarity, while facial appearance had a main effect on familiarity. However, these variables had no effects on attraction. As discussed in the operational definitions, attraction is based on a person’s liking for the other, and perceived liking in the initial stage of interaction may lead to feelings of attraction [58]. Our results also show that among the constructs, perceived attraction and liking have the highest correlation (r = 0.695, *p* < 0.01).

**Figure 13:**
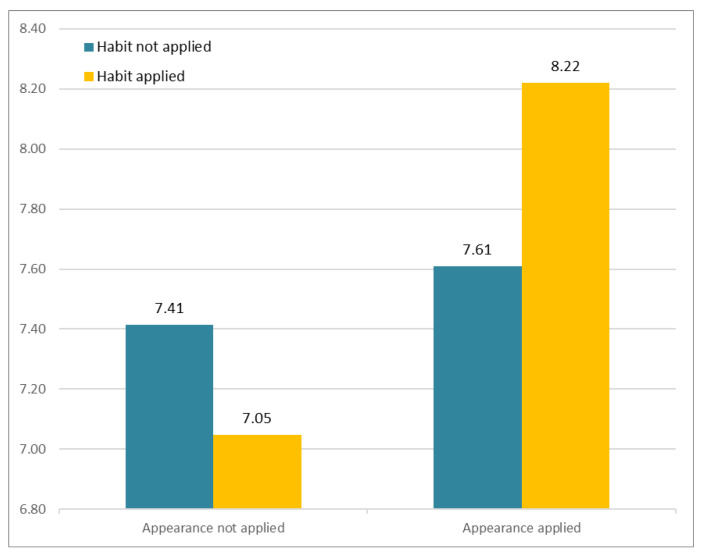
Subjective appraisal of the sum of similarity and liking.

However, attraction is a much larger and multifaceted construct [63]. Based on the pioneering work by Byrne [64], both perceived similarity and liking lead to attraction, and many researchers have attempted to understand the exact interplay and different weights of the two on attraction [44]. Therefore, we conducted a two-way ANOVA on the sum of perceived liking and similarity (i.e., data categorization) of the four avatar conditions (see Figure 13). The *Y*-axis indicates the addition of the Likert ratings of perceived liking and similarity.

The results showed that Facial Habit had a significant main effect, F(1, 81) = 4.836, *p* < 0.05, whereas Facial Appearance had no significant main effect, F(1, 81) = 0.610, *p* > 0.69. Furthermore, there was no significant interaction between Facial Habit × Facial Appearance, F(1, 163) = 2.467, *p* > 0.12.

The research investigated the participant’s social perception (similarity, familiarity, attraction, liking, and involvement) of virtual avatars engineered with the participant’s unique facial signature (facial appearance, facial habit). In summary, the participants perceived significant similarity to an avatar with habitual expression applied compared to the avatar that did not (*p* < 0.05). In addition, habitual expressions also significantly affected the sum of perceived similarity and perceived liking (*p* < 0.05). The participants perceived familiarity with the avatar with facial appearance applied compared to the avatar that did not (*p* < 0.05).

## 4. Discussion and Conclusions

To our knowledge, this is the first research to reveal that participants can perceive similarity to a virtual human that had their characteristic facial movements (i.e., habitual pattern), which has significant implications for the design of virtual agents. The virtual human community had long researched the effects of virtual agent realism. The consensus is that behavioral realism is more critical than visual realism in eliciting believability [27]. The suspension of disbelief refers to the deliberate avoidance of critical thinking, whereas a reality check involves deciding what is possible or not in the real world [65,66]. Thus, behavioral realism is more socially engaging and believable than visual realism [27].

In the context of this study, the effect of perceived similarity of a virtual agent to oneself is consistent with research findings on believability. Specifically, while participants did not perceive similarity in virtual avatars to which their facial appearance were applied (i.e., visual realism), they perceived similarity in virtual avatars to which their facial habits were applied (i.e., behavioral realism). This implies that designs may go beyond anthropomorphic design. For example, future research may conduct studies using animal-inspired avatars with facial features (e.g., eyes) and see if participants can perceive similarity to these avatars when their facial movements are applied.

There is much empirical evidence that similarity, as a social construct, elicits attraction [44], and this relationship is regarded as “one of the most robust relationships in all of the behavioral sciences (p. 281)” [67]. Researchers found a positive linear relationship between similarity and attraction (i.e., the law of attraction) [68]. However, the various virtual avatars had no significant effect on attraction. This may be due to interaction being limited to one-time mere exposure. We purposely limited interaction to exclude variables (e.g., perception of personality) that may influence the perceived measures, which may have been brought on by prolonged interaction. Perceived similarity is influenced not only by physical appearance [69] but also attitude [70] and personality [71]. Future studies may add a persona to the virtual avatar to test the complexities of perceived similarity.

The study’s limitation in understanding the effects of an individuated avatar on attraction is apparent. Since perceived attraction is a multifaceted construct, it typically requires more interaction, building up from initial liking [58]. Future studies may investigate the degree of attraction as a function of time or when participants interact with the individuated virtual avatar. The perceived relationship also influences attraction; thus, future studies need to address the relationship between the avatar (e.g., companion, butler, assistant) and the participant carefully.

Nevertheless, through data categorization, we found that habitual expressions had a main effect on the sum of perceived similarity and perceived liking (*p* < 0.05). Since the interplay between perceived similarity and liking leads to attraction [64], these results suggest that an individuated avatar may elicit attraction with prolonged interaction.

Additionally, the individualized virtual avatars had no significant effect on perceived involvement. Although we provided the context that the virtual agent would be used as part of a profile for a social networking service, we also acknowledge that many users do not use profiles similar to their appearance. Future studies should cluster the participants based on who use or intend to use avatars with a similar appearance as an alter ego and assess their perceived responses accordingly.

The perceived familiarity with a virtual avatar to which the participant’s facial appearance was applied may be due to the participant’s repetitive exposure to their reflections in mirrors or still photos of themselves. Repetitive exposure elicits familiarity [13]. On the other hand, people may not be familiar with their habitual expressions during various emotional states.

Finally, the study is limited in that the virtual avatars were designed based on only positive emotional expressions. Future research on individualized virtual avatars should also include negative or complex emotions.

## Figures and Tables

**Table 1:** Research hypotheses.

Research Hypotheses	
H1	A virtual avatar that displays the participant’s habitual expressions will elicit the following perceived social constructs more than a virtual avatar that does not: Perceived similarity Perceived familiarity Perceived attraction Perceived liking Perceived involvement
H2	A virtual avatar that has a similar facial appearance to the participant will elicit the following perceived social constructs more than a virtual avatar that does not: Perceived similarity Perceived familiarity Perceived attraction Perceived liking Perceived involvement
H3	There is an interaction between the participant’s habitual expressions and facial appearance.

**Table 2:** The blend shape type based on the virtual avatar’s AU and facial appearance.

Blend Shape	Description	Muscular Basis
AU1	Inner brow raiser	Frontalis, Pars medialis
AU2	Outer brow raiser	Frontalis, Pars lateralis
AU4	Brow lowerer	Depressor glabellae, Depressor supercilli, Corrugator supercilli
AU5	Upper lid raiser	Levator palpebrae superioris
AU6	Cheek raiser	Orbicularis oculi, Pars orbitalis
AU7	Lid tightener	Orbicularis oculi, Pars palpebralis
AU9	Nose wrinkler	Levator labii superioris alaeque nasi
AU10	Upper lip raiser	Levator labii superioris, Caput infraorbitalis
AU12	Lip corner puller	Zygomaticus major
AU14	Dimpler	Buccinator
AU15	Lip corner depressor	Depressor anguli oris (Triangularis)
AU17	Chin raiser	Mentalis
AU20	Lip stretcher	Risorius
AU23	Lip tightener	Orbicularis oris
AU25	Lips part	Depressor labii, Relaxation of mentalis (AU17), Orbicularis oris
AU26	Jaw drop	Masseter, Temporal and Internal pterygoid relaxed
AU28	Lip suck	Orbicularis oris
AU45	Blink	Relaxation of levator palpebrae and Contraction of orbicularis oculi, Pars palpebralis.
Shape1	Expansion of the lower jaw bone	Mandible ramus extension
Shape2	Contraction of the lower jaw bone	Mandible ramus compression
Shape3	Expansion of the lower jaw	Chin extension
Shape4	Contraction of the lower jaw	Chin compression

**Table 3:** The operational definition of the social constructs of interest.

Social Construct	Operational Definition
Similarity	The degree to which the participant *believes* the virtual avatar’s appearance is similar to themselves.
Familiarity	The degree to which the participant is *familiar* with the virtual avatar’s appearance.
Attraction	The degree to which the participant is *attracted to* the virtual avatar.
Liking	The degree to which the participant *likes or dislikes* the virtual avatar.
Involvement	The degree to which the participant *relates to* or *empathizes with* the virtual avatar.

**Table 4:** The Pearson correlation coefficients between perceived social constructs (*n* = 164, *p* *** < 0.01).

	Similarity	Familiarity	Attraction	Liking	Involvement
**Similarity**		0.425 ***	0.304 ***	0.432 ***	0.376 ***
**Familiarity**			0.597 ***	0.564 ***	0.500 ***
**Attraction**				0.659 ***	0.588 ***
**Liking**					0.499 ***
**Involvement**					
